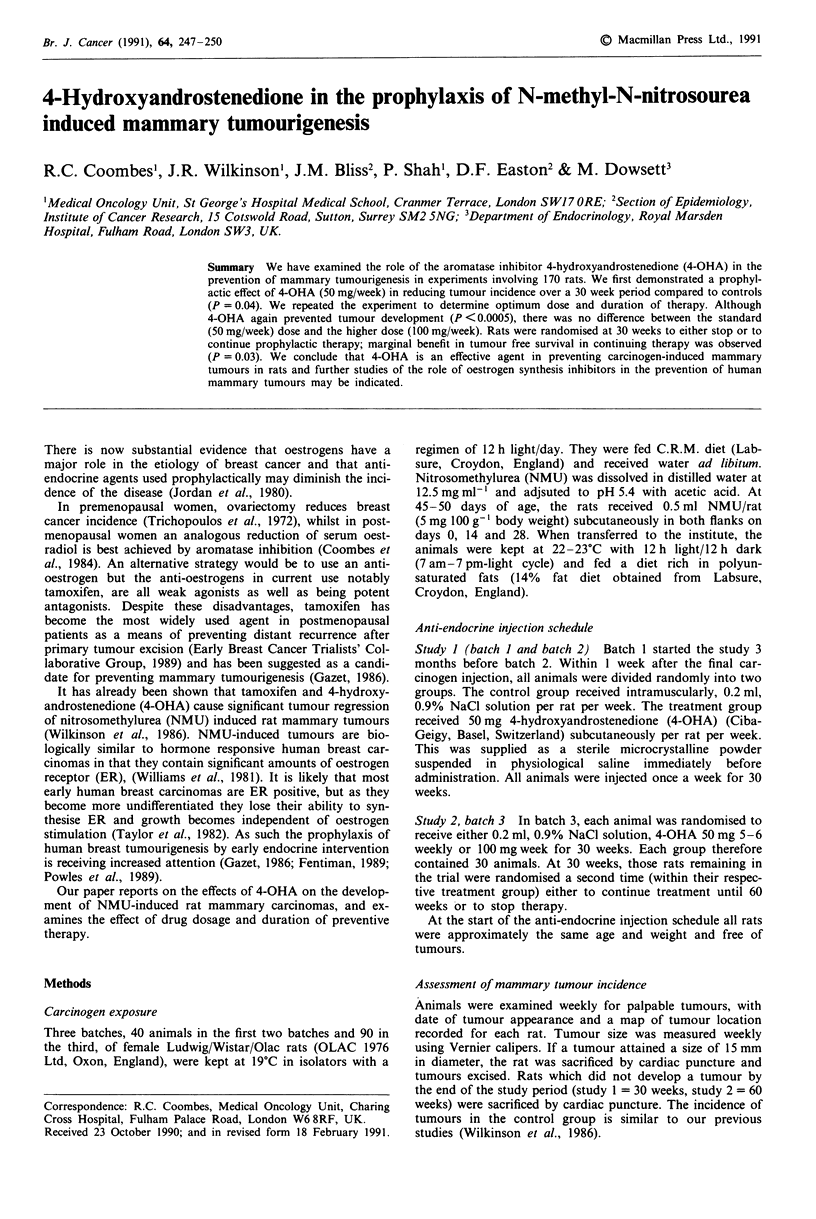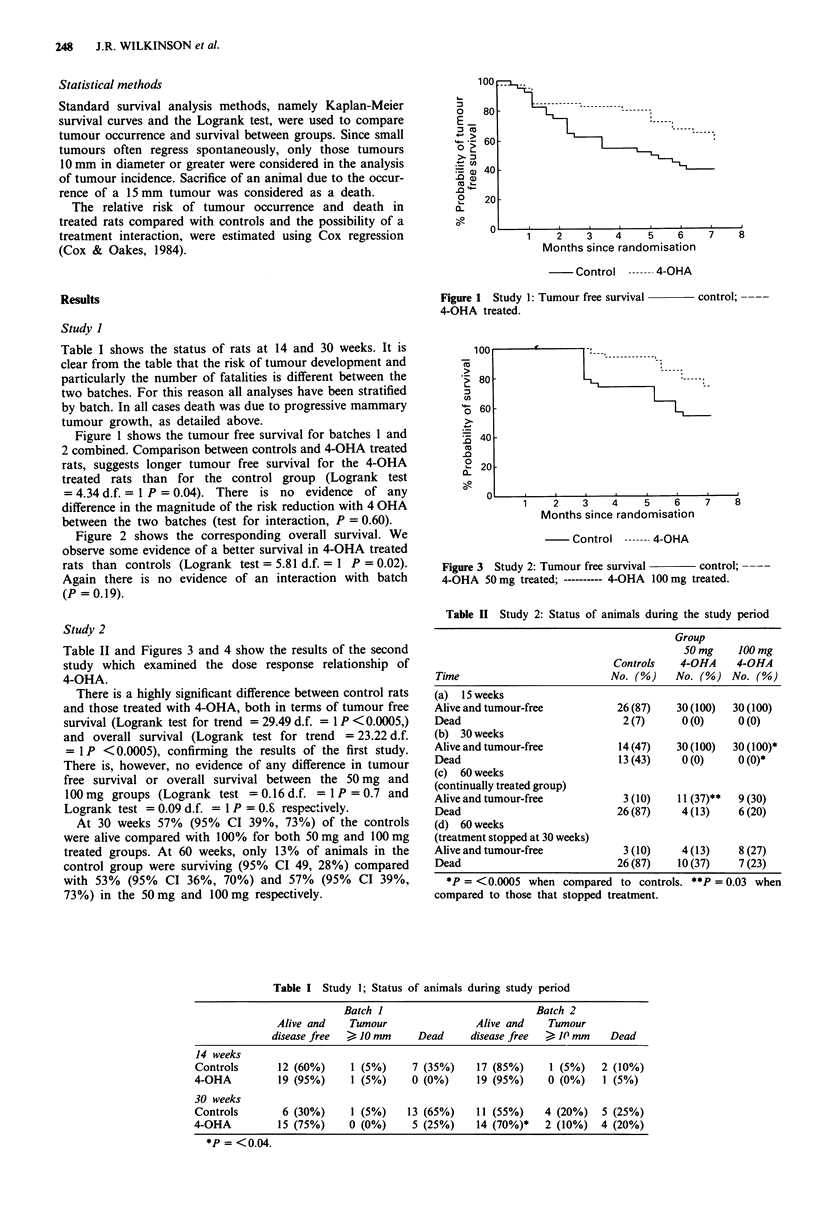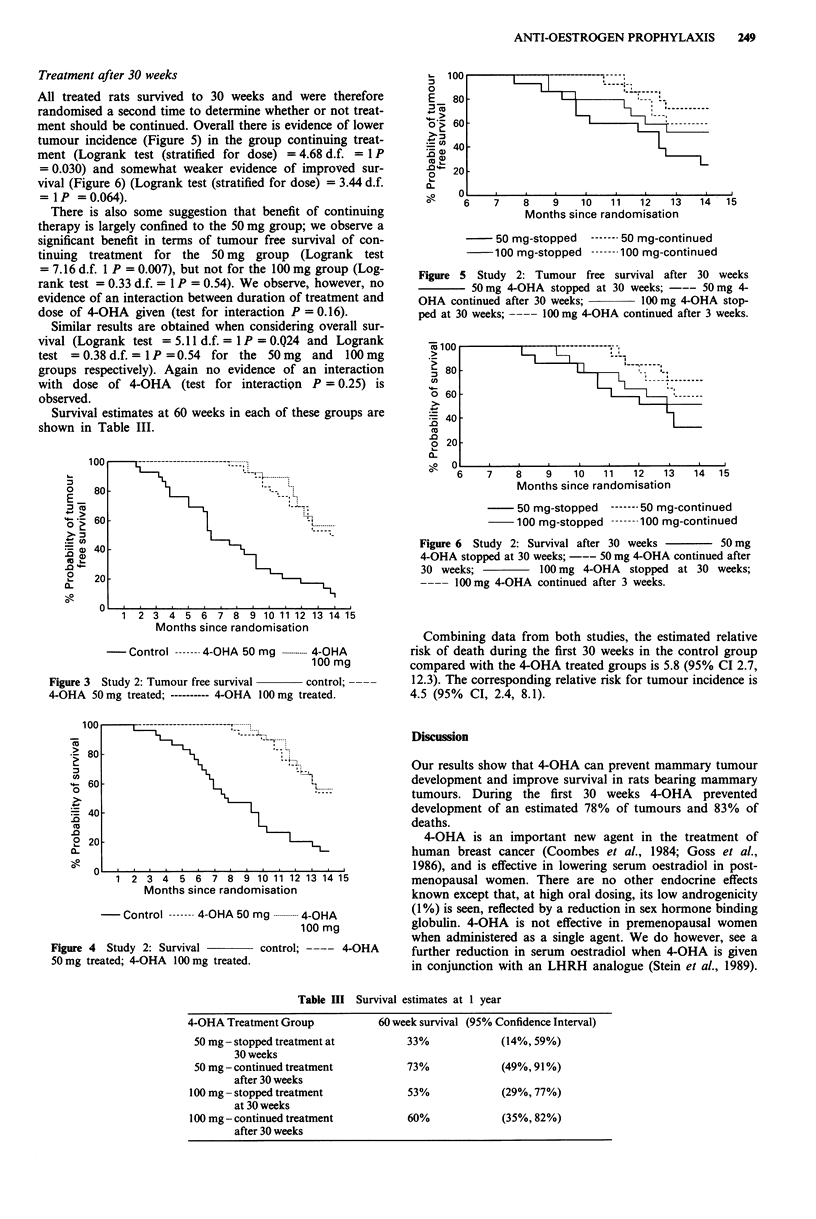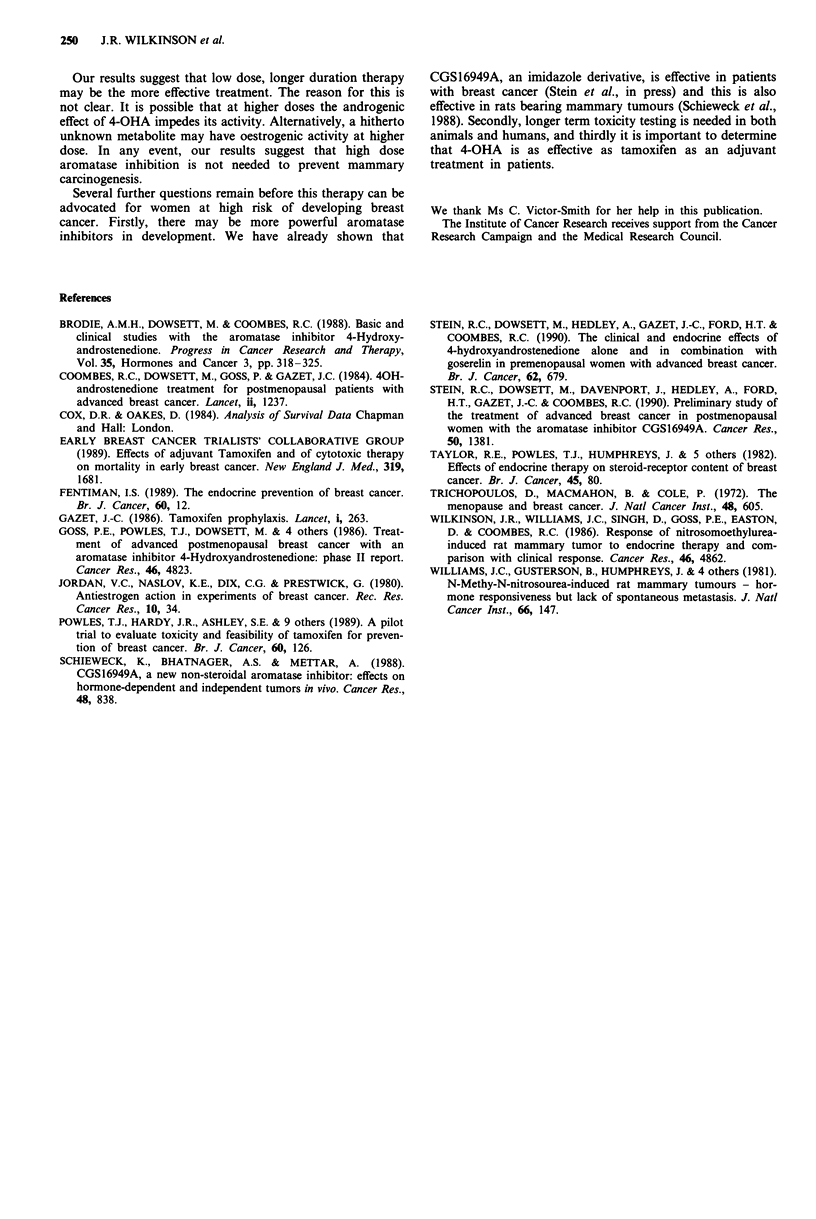# 4-Hydroxyandrostenedione in the prophylaxis of N-methyl-N-nitrosourea induced mammary tumourigenesis.

**DOI:** 10.1038/bjc.1991.285

**Published:** 1991-08

**Authors:** R. C. Coombes, J. R. Wilkinson, J. M. Bliss, P. Shah, D. F. Easton, M. Dowsett

**Affiliations:** Medical Oncology Unit, St George's Hospital Medical School, London, UK.

## Abstract

We have examined the role of the aromatase inhibitor 4-hydroxyandrostenedione (4-OHA) in the prevention of mammary tumourigenesis in experiments involving 170 rats. We first demonstrated a prophylactic effect of 4-OHA (50 mg/week) in reducing tumour incidence over a 30 week period compared to controls (P = 0.04). We repeated the experiment to determine optimum dose and duration of therapy. Although 4-OHA again prevented tumour development (P less than 0.0005), there was no difference between the standard (50 mg/week) dose and the higher dose (100 mg/week). Rats were randomised at 30 weeks to either stop or to continue prophylactic therapy; marginal benefit in tumour free survival in continuing therapy was observed (P = 0.03). We conclude that 4-OHA is an effective agent in preventing carcinogen-induced mammary tumours in rats and further studies of the role of oestrogen synthesis inhibitors in the prevention of human mammary tumours may be indicated.


Br. J. Cancer (1991), 64, 247–250 © Macmillan Press Ltd., 1991

### 4-Hydroxyandrostenedione in the prophylaxis of N-methyl-N-nitrosourea induced mammary tumourigenesis

R.C. Coombes[1], J.R. Wilkinson[1], J.M. Bliss[2], P. Shah[1], D.F. Easton[2] & M. Dowsett[3]

[1]*Medical Oncology Unit, St George's Hospital Medical School, Cranmer Terrace, London SW17 0RE;* [2]*Section of Epidemiology, Institute of Cancer Research, 15 Cotswold Road, Sutton, Surrey SM2 5NG;* [3]*Department of Endocrinology, Royal Marsden Hospital, Fulham Road, London SW3, UK.*

**Summary** We have examined the role of the aromatase inhibitor 4-hydroxyandrostenedione (4-OHA) in the prevention of mammary tumourigenesis in experiments involving 170 rats. We first demonstrated a prophylactic effect of 4-OHA (50 mg/week) in reducing tumour incidence over a 30 week period compared to controls ($P = 0.04$). We repeated the experiment to determine optimum dose and duration of therapy. Although 4-OHA again prevented tumour development ($P < 0.0005$), there was no difference between the standard (50 mg/week) dose and the higher dose (100 mg/week). Rats were randomised at 30 weeks to either stop or to continue prophylactic therapy; marginal benefit in tumour free survival in continuing therapy was observed ($P = 0.03$). We conclude that 4-OHA is an effective agent in preventing carcinogen-induced mammary tumours in rats and further studies of the role of oestrogen synthesis inhibitors in the prevention of human mammary tumours may be indicated.

There is now substantial evidence that oestrogens have a major role in the etiology of breast cancer and that anti-endocrine agents used prophylactically may diminish the incidence of the disease (Jordan *et al.*, 1980).

In premenopausal women, ovariectomy reduces breast cancer incidence (Trichopoulos *et al.*, 1972), whilst in post-menopausal women an analogous reduction of serum oestradiol is best achieved by aromatase inhibition (Coombes *et al.*, 1984). An alternative strategy would be to use an anti-oestrogen but the anti-oestrogens in current use notably tamoxifen, are all weak agonists as well as being potent antagonists. Despite these disadvantages, tamoxifen has become the most widely used agent in postmenopausal patients as a means of preventing distant recurrence after primary tumour excision (Early Breast Cancer Trialists' Collaborative Group, 1989) and has been suggested as a candidate for preventing mammary tumourigenesis (Gazet, 1986).

It has already been shown that tamoxifen and 4-hydroxyandrostenedione (4-OHA) cause significant tumour regression of nitrosomethylurea (NMU) induced rat mammary tumours (Wilkinson *et al.*, 1986). NMU-induced tumours are biologically similar to hormone responsive human breast carcinomas in that they contain significant amounts of oestrogen receptor (ER), (Williams *et al.*, 1981). It is likely that most early human breast carcinomas are ER positive, but as they become more undifferentiated they lose their ability to synthesise ER and growth becomes independent of oestrogen stimulation (Taylor *et al.*, 1982). As such the prophylaxis of human breast tumourigenesis by early endocrine intervention is receiving increased attention (Gazet, 1986; Fentiman, 1989; Powles *et al.*, 1989).

Our paper reports on the effects of 4-OHA on the development of NMU-induced rat mammary carcinomas, and examines the effect of drug dosage and duration of preventive therapy.

### Methods

### *Carcinogen exposure*

Three batches, 40 animals in the first two batches and 90 in the third, of female Ludwig/Wistar/Olac rats (OLAC 1976 Ltd, Oxon, England), were kept at 19°C in isolators with a regimen of 12 h light/day. They were fed C.R.M. diet (Labsure, Croydon, England) and received water *ad libitum*. Nitrosomethylurea (NMU) was dissolved in distilled water at 12.5 mg ml$^{-1}$ and adjsuted to pH 5.4 with acetic acid. At 45–50 days of age, the rats received 0.5 ml NMU/rat (5 mg 100 g$^{-1}$ body weight) subcutaneously in both flanks on days 0, 14 and 28. When transferred to the institute, the animals were kept at 22–23°C with 12 h light/12 h dark (7 am–7 pm-light cycle) and fed a diet rich in polyunsaturated fats (14% fat diet obtained from Labsure, Croydon, England).

### *Anti-endocrine injection schedule*

*Study 1 (batch 1 and batch 2)* Batch 1 started the study 3 months before batch 2. Within 1 week after the final carcinogen injection, all animals were divided randomly into two groups. The control group received intramuscularly, 0.2 ml, 0.9% NaCl solution per rat per week. The treatment group received 50 mg 4-hydroxyandrostenedione (4-OHA) (Ciba-Geigy, Basel, Switzerland) subcutaneously per rat per week. This was supplied as a sterile microcrystalline powder suspended in physiological saline immediately before administration. All animals were injected once a week for 30 weeks.

*Study 2, batch 3* In batch 3, each animal was randomised to receive either 0.2 ml, 0.9% NaCl solution, 4-OHA 50 mg 5–6 weekly or 100 mg week for 30 weeks. Each group therefore contained 30 animals. At 30 weeks, those rats remaining in the trial were randomised a second time (within their respective treatment group) either to continue treatment until 60 weeks or to stop therapy.

At the start of the anti-endocrine injection schedule all rats were approximately the same age and weight and free of tumours.

### *Assessment of mammary tumour incidence*

Animals were examined weekly for palpable tumours, with date of tumour appearance and a map of tumour location recorded for each rat. Tumour size was measured weekly using Vernier calipers. If a tumour attained a size of 15 mm in diameter, the rat was sacrificed by cardiac puncture and tumours excised. Rats which did not develop a tumour by the end of the study period (study 1 = 30 weeks, study 2 = 60 weeks) were sacrificed by cardiac puncture. The incidence of tumours in the control group is similar to our previous studies (Wilkinson *et al.*, 1986).

Correspondence: R.C. Coombes, Medical Oncology Unit, Charing Cross Hospital, Fulham Palace Road, London W6 8RF, UK.
Received 23 October 1990; and in revised form 18 February 1991.

*Statistical methods*

Standard survival analysis methods, namely Kaplan-Meier survival curves and the Logrank test, were used to compare tumour occurrence and survival between groups. Since small tumours often regress spontaneously, only those tumours 10 mm in diameter or greater were considered in the analysis of tumour incidence. Sacrifice of an animal due to the occurrence of a 15 mm tumour was considered as a death.

The relative risk of tumour occurrence and death in treated rats compared with controls and the possibility of a treatment interaction, were estimated using Cox regression (Cox & Oakes, 1984).

### Results

*Study 1*

Table I shows the status of rats at 14 and 30 weeks. It is clear from the table that the risk of tumour development and particularly the number of fatalities is different between the two batches. For this reason all analyses have been stratified by batch. In all cases death was due to progressive mammary tumour growth, as detailed above.

Figure 1 shows the tumour free survival for batches 1 and 2 combined. Comparison between controls and 4-OHA treated rats, suggests longer tumour free survival for the 4-OHA treated rats than for the control group (Logrank test = 4.34 d.f. = 1 $P = 0.04$). There is no evidence of any difference in the magnitude of the risk reduction with 4 OHA between the two batches (test for interaction, $P = 0.60$).

Figure 2 shows the corresponding overall survival. We observe some evidence of a better survival in 4-OHA treated rats than controls (Logrank test = 5.81 d.f. = 1 $P = 0.02$). Again there is no evidence of an interaction with batch ($P = 0.19$).

*Study 2*

Table II and Figures 3 and 4 show the results of the second study which examined the dose response relationship of 4-OHA.

There is a highly significant difference between control rats and those treated with 4-OHA, both in terms of tumour free survival (Logrank test for trend = 29.49 d.f. = 1 $P < 0.0005$,) and overall survival (Logrank test for trend = 23.22 d.f. = 1 $P < 0.0005$), confirming the results of the first study. There is, however, no evidence of any difference in tumour free survival or overall survival between the 50 mg and 100 mg groups (Logrank test = 0.16 d.f. = 1 $P = 0.7$ and Logrank test = 0.09 d.f. = 1 $P = 0.8$ respectively.

At 30 weeks 57% (95% CI 39%, 73%) of the controls were alive compared with 100% for both 50 mg and 100 mg treated groups. At 60 weeks, only 13% of animals in the control group were surviving (95% CI 49, 28%) compared with 53% (95% CI 36%, 70%) and 57% (95% CI 39%, 73%) in the 50 mg and 100 mg respectively.

**Figure 1** Study 1: Tumour free survival ——— control; ---- 4-OHA treated.

**Figure 3** Study 2: Tumour free survival ——— control; ---- 4-OHA 50 mg treated; --------- 4-OHA 100 mg treated.

**Table II** Study 2: Status of animals during the study period

| Time | Controls No. (%) | Group 50 mg 4-OHA No. (%) | 100 mg 4-OHA No. (%) |
|---|---|---|---|
| (a) 15 weeks | | | |
| Alive and tumour-free | 26 (87) | 30 (100) | 30 (100) |
| Dead | 2 (7) | 0 (0) | 0 (0) |
| (b) 30 weeks | | | |
| Alive and tumour-free | 14 (47) | 30 (100) | 30 (100)* |
| Dead | 13 (43) | 0 (0) | 0 (0)* |
| (c) 60 weeks (continually treated group) | | | |
| Alive and tumour-free | 3 (10) | 11 (37)** | 9 (30) |
| Dead | 26 (87) | 4 (13) | 6 (20) |
| (d) 60 weeks (treatment stopped at 30 weeks) | | | |
| Alive and tumour-free | 3 (10) | 4 (13) | 8 (27) |
| Dead | 26 (87) | 10 (37) | 7 (23) |

*$P = < 0.0005$ when compared to controls. **$P = 0.03$ when compared to those that stopped treatment.

**Table I** Study 1; Status of animals during study period

| | Batch 1 Alive and disease free | Tumour ≥ 10 mm | Dead | Batch 2 Alive and disease free | Tumour ≥ 10 mm | Dead |
|---|---|---|---|---|---|---|
| *14 weeks* | | | | | | |
| Controls | 12 (60%) | 1 (5%) | 7 (35%) | 17 (85%) | 1 (5%) | 2 (10%) |
| 4-OHA | 19 (95%) | 1 (5%) | 0 (0%) | 19 (95%) | 0 (0%) | 1 (5%) |
| *30 weeks* | | | | | | |
| Controls | 6 (30%) | 1 (5%) | 13 (65%) | 11 (55%) | 4 (20%) | 5 (25%) |
| 4-OHA | 15 (75%) | 0 (0%) | 5 (25%) | 14 (70%)* | 2 (10%) | 4 (20%) |

*$P = < 0.04$.

*Treatment after 30 weeks*

All treated rats survived to 30 weeks and were therefore randomised a second time to determine whether or not treatment should be continued. Overall there is evidence of lower tumour incidence (Figure 5) in the group continuing treatment (Logrank test (stratified for dose) = 4.68 d.f. = 1 $P$ = 0.030) and somewhat weaker evidence of improved survival (Figure 6) (Logrank test (stratified for dose) = 3.44 d.f. = 1 $P$ = 0.064).

There is also some suggestion that benefit of continuing therapy is largely confined to the 50 mg group; we observe a significant benefit in terms of tumour free survival of continuing treatment for the 50 mg group (Logrank test = 7.16 d.f. 1 $P$ = 0.007), but not for the 100 mg group (Logrank test = 0.33 d.f. = 1 $P$ = 0.54). We observe, however, no evidence of an interaction between duration of treatment and dose of 4-OHA given (test for interaction $P$ = 0.16).

Similar results are obtained when considering overall survival (Logrank test = 5.11 d.f. = 1 $P$ = 0.024 and Logrank test = 0.38 d.f. = 1 $P$ = 0.54 for the 50 mg and 100 mg groups respectively). Again no evidence of an interaction with dose of 4-OHA (test for interaction $P$ = 0.25) is observed.

Survival estimates at 60 weeks in each of these groups are shown in Table III.

**Figure 3** Study 2: Tumour free survival ———— control; ---- 4-OHA 50 mg treated; --------- 4-OHA 100 mg treated.

**Figure 4** Study 2: Survival ———— control; ---- 4-OHA 50 mg treated; 4-OHA 100 mg treated.

**Figure 5** Study 2: Tumour free survival after 30 weeks ———— 50 mg 4-OHA stopped at 30 weeks; ---- 50 mg 4-OHA continued after 30 weeks; ———— 100 mg 4-OHA stopped at 30 weeks; ---- 100 mg 4-OHA continued after 3 weeks.

**Figure 6** Study 2: Survival after 30 weeks ———— 50 mg 4-OHA stopped at 30 weeks; ---- 50 mg 4-OHA continued after 30 weeks; ———— 100 mg 4-OHA stopped at 30 weeks; ---- 100 mg 4-OHA continued after 3 weeks.

Combining data from both studies, the estimated relative risk of death during the first 30 weeks in the control group compared with the 4-OHA treated groups is 5.8 (95% CI 2.7, 12.3). The corresponding relative risk for tumour incidence is 4.5 (95% CI, 2.4, 8.1).

### Discussion

Our results show that 4-OHA can prevent mammary tumour development and improve survival in rats bearing mammary tumours. During the first 30 weeks 4-OHA prevented development of an estimated 78% of tumours and 83% of deaths.

4-OHA is an important new agent in the treatment of human breast cancer (Coombes *et al.*, 1984; Goss *et al.*, 1986), and is effective in lowering serum oestradiol in postmenopausal women. There are no other endocrine effects known except that, at high oral dosing, its low androgenicity (1%) is seen, reflected by a reduction in sex hormone binding globulin. 4-OHA is not effective in premenopausal women when administered as a single agent. We do however, see a further reduction in serum oestradiol when 4-OHA is given in conjunction with an LHRH analogue (Stein *et al.*, 1989).

**Table III** Survival estimates at 1 year

| 4-OHA Treatment Group | 60 week survival | (95% Confidence Interval) |
|---|---|---|
| 50 mg – stopped treatment at 30 weeks | 33% | (14%, 59%) |
| 50 mg – continued treatment after 30 weeks | 73% | (49%, 91%) |
| 100 mg – stopped treatment at 30 weeks | 53% | (29%, 77%) |
| 100 mg – continued treatment after 30 weeks | 60% | (35%, 82%) |

Our results suggest that low dose, longer duration therapy may be the more effective treatment. The reason for this is not clear. It is possible that at higher doses the androgenic effect of 4-OHA impedes its activity. Alternatively, a hitherto unknown metabolite may have oestrogenic activity at higher dose. In any event, our results suggest that high dose aromatase inhibition is not needed to prevent mammary carcinogenesis.

Several further questions remain before this therapy can be advocated for women at high risk of developing breast cancer. Firstly, there may be more powerful aromatase inhibitors in development. We have already shown that CGS16949A, an imidazole derivative, is effective in patients with breast cancer (Stein et al., in press) and this is also effective in rats bearing mammary tumours (Schieweck et al., 1988). Secondly, longer term toxicity testing is needed in both animals and humans, and thirdly it is important to determine that 4-OHA is as effective as tamoxifen as an adjuvant treatment in patients.

We thank Ms C. Victor-Smith for her help in this publication.

The Institute of Cancer Research receives support from the Cancer Research Campaign and the Medical Research Council.

### References

BRODIE, A.M.H., DOWSETT, M. & COOMBES, R.C. (1988). Basic and clinical studies with the aromatase inhibitor 4-Hydroxy-androstenedione. *Progress in Cancer Research and Therapy*, Vol. **35**, Hormones and Cancer 3, pp. 318–325.

COOMBES, R.C., DOWSETT, M., GOSS, P. & GAZET, J.C. (1984). 4OH-androstenedione treatment for postmenopausal patients with advanced breast cancer. *Lancet*, **ii**, 1237.

COX, D.R. & OAKES, D. (1984). *Analysis of Survival Data* Chapman and Hall: London.

EARLY BREAST CANCER TRIALISTS' COLLABORATIVE GROUP (1989). Effects of adjuvant Tamoxifen and of cytotoxic therapy on mortality in early breast cancer. *New England J. Med.*, **319**, 1681.

FENTIMAN, I.S. (1989). The endocrine prevention of breast cancer. *Br. J. Cancer*, **60**, 12.

GAZET, J.-C. (1986). Tamoxifen prophylaxis. *Lancet*, **i**, 263.

GOSS, P.E., POWLES, T.J., DOWSETT, M. & 4 others (1986). Treatment of advanced postmenopausal breast cancer with an aromatase inhibitor 4-Hydroxyandrostenedione: phase II report. *Cancer Res.*, **46**, 4823.

JORDAN, V.C., NASLOV, K.E., DIX, C.G. & PRESTWICK, G. (1980). Antiestrogen action in experiments of breast cancer. *Rec. Res. Cancer Res.*, **10**, 34.

POWLES, T.J., HARDY, J.R., ASHLEY, S.E. & 9 others (1989). A pilot trial to evaluate toxicity and feasibility of tamoxifen for prevention of breast cancer. *Br. J. Cancer*, **60**, 126.

SCHIEWECK, K., BHATNAGER, A.S. & METTAR, A. (1988). CGS16949A, a new non-steroidal aromatase inhibitor: effects on hormone-dependent and independent tumors *in vivo*. *Cancer Res.*, **48**, 838.

STEIN, R.C., DOWSETT, M., HEDLEY, A., GAZET, J.-C., FORD, H.T. & COOMBES, R.C. (1990). The clinical and endocrine effects of 4-hydroxyandrostenedione alone and in combination with goserelin in premenopausal women with advanced breast cancer. *Br. J. Cancer*, **62**, 679.

STEIN, R.C., DOWSETT, M., DAVENPORT, J., HEDLEY, A., FORD, H.T., GAZET, J.-C. & COOMBES, R.C. (1990). Preliminary study of the treatment of advanced breast cancer in postmenopausal women with the aromatase inhibitor CGS16949A. *Cancer Res.*, **50**, 1381.

TAYLOR, R.E., POWLES, T.J., HUMPHREYS, J. & 5 others (1982). Effects of endocrine therapy on steroid-receptor content of breast cancer. *Br. J. Cancer*, **45**, 80.

TRICHOPOULOS, D., MACMAHON, B. & COLE, P. (1972). The menopause and breast cancer. *J. Natl Cancer Inst.*, **48**, 605.

WILKINSON, J.R., WILLIAMS, J.C., SINGH, D., GOSS, P.E., EASTON, D. & COOMBES, R.C. (1986). Response of nitrosomoethylurea-induced rat mammary tumor to endocrine therapy and comparison with clinical response. *Cancer Res.*, **46**, 4862.

WILLIAMS, J.C., GUSTERSON, B., HUMPHREYS, J. & 4 others (1981). N-Methy-N-nitrosourea-induced rat mammary tumours – hormone responsiveness but lack of spontaneous metastasis. *J. Natl Cancer Inst.*, **66**, 147.